# Total Glucosides of Paeony Ameliorate Myocardial Injury in Chronic Heart Failure Rats by Suppressing PARP-1

**DOI:** 10.1007/s12265-023-10440-3

**Published:** 2023-10-13

**Authors:** Wenjuan Wei, Caiyan Li, Baoyong Zhang, Deyun Huang, Zheming Li, Jiaer Gao

**Affiliations:** 1 Department of Cardiology, The First People’s Hospital of Xiaoshan District, No. 199, Shixin Nan Road, Xiaoshan District, Hangzhou, 311200 Zhejiang China; 2 College of Pharmacy, Hangzhou Medical College, No. 481, Binwen Road, Binjiang District, Hangzhou, 310053 Zhejiang China

**Keywords:** Chronic heart failure, Total glucosides of paeony, Inflammatory response, PARP-1, NF-κB

## Abstract

Total glucosides of paeony (TGP) have a potential protective effect on chronic heart failure (CHF) rats, but the mechanism remains unclear. PARP inhibition prevents the decrease in myocardial contractility. Therefore, we aim to investigate the effects and mechanisms of TGP on CHF and the role of PARP-1 in CHF. Left anterior descending ligation rats and adriamycin-treated H9C9 cells were used as CHF models, and captopril as a positive control for in vivo experiments. We found that TGP alleviated myocardial remodeling and improved cardiac morphology and function. TGP also reduced myocardial apoptosis and autophagy, decreased inflammatory factor release, and inhibited the PARP-1 and NF-κB proteins. Through cell transfection, we found that PAPR-1 knockdown inhibited NF-κB nuclear translocation. Additionally, TGP inhibited apoptosis, autophagy, and inflammation in CHF cells, while PARP-1 overexpression partially antagonized them. In conclusion, TGP has the potential to improve CHF and PARP-1 may be a potential target.

**Graphical Abstract:**

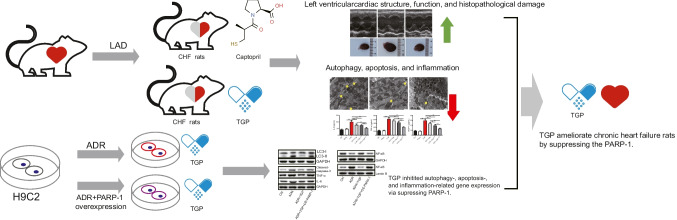

**Supplementary Information:**

The online version contains supplementary material available at 10.1007/s12265-023-10440-3.

## Introduction

Chronic heart failure (CHF) is a late-stage manifestation of various chronic cardio-cerebrovascular disorders [1, 2]. With the increasing aging population and rising incidence of chronic diseases such as diabetes, dyslipidemia, hypertension, coronary atherosclerotic heart disease, and obesity, the prevalence of CHF continues to rise [3, 4]. Although new medical technologies make it possible to extend the survival time of these patients, the majority of individuals eventually develop heart failure. CHF patients’ hearts exhibit aberrant changes in shape and function, often with ventricular systolic or diastolic dysfunction [5]. According to a China heart failure prevalence analysis, the prevalence of heart failure in urban China is 275 per 100,000 person-years [6]. The Chinese Heart Failure Registration Study reported that the mortality of patients with heart failure is 4.1% [7]. While in-hospital mortality has decreased significantly, the rate of rehospitalization continues to rise and patients have a poor prognosis [8]. Therefore, scientists aim to enhance patients’ quality of life, reduce mortality and rehospitalization rates, and improve long-term prognosis. To achieve these goals, more efficient prevention and treatment strategies are necessary.

Ventricular remodeling is considered the primary cause of CHF [9], which can be triggered by various factors including cardiac hypertrophy, myocyte apoptosis and autophagy, collagen deposition in the extracellular matrix, fibrosis, changes in metabolic and electrophysiological characteristics, and inflammation [10, 11]. Angiotensin-converting enzyme inhibitors, such as captopril, are a first-line therapy for patients with a reduced ejection fraction [12], as they are known to reduce inflammation and apoptosis of myocardial cells [13, 14]. Captopril has a dual effect of inhibiting and inducing cell apoptosis, while also reversing the inflammatory response mediated by autophagy according to fundamental investigations [15]. Autophagy and apoptosis are two distinct mechanisms of programmed cell death that regulate the cell viability and death. While apoptosis can protect cells, tissues, and organs from necrosis-induced inflammatory responses, excessive apoptosis can lead to tissue damage [16]. Autophagy, on the other hand, is the primary survival mechanism for cells in times of stress, allowing them to recycle cellular structures and create energy substances; however, excessive activation of autophagy can also cause damage to intracellular molecules and organelles, leading to organ injury [17]. It is evident that both cell apoptosis and autophagy, which are related to inflammation, may be key mechanisms involved in myocardial injury.

Total glucosides of paeony (TGP) possess well-demonstrable antioxidant, anti-inflammatory, and immunomodulatory properties and have shown potential as a treatment for cardiovascular disease [18]. Evidence suggests that TGP can effectively reduce inflammation and protect heart failure in rats suggesting its potential value for clinical applications [19]. TGP has been used to treat rheumatoid arthritis as an anti-inflammatory immunomodulator agent. More recently, it has been found to inhibit myocardial inflammation, counteract arteriosclerosis, and enhance myocardial remodeling [20]. Clinical investigations have demonstrated that TGP effectively suppresses inflammation in stable CHF patients by reducing the release of proinflammatory cytokines, including tumor necrosis factor-α (TNF-α) and interleukin-6 (IL-6) [20]. These cytokines play a critical role in the body’s inflammatory response. Research has linked poly(ADP-ribose) polymerase-1 (PARP-1), a key gene for many TCM [21–23], and is involved in autophagy, to the expression of TNF-α and IL-6 [24], as well as oxidative stress and autophagy in vascular smooth muscle cells [25]. In animal models of CHF, inhibition of PARP leads to significant improvement in left ventricular systolic pressure, ventricular contractility, and relaxation [26]. It also reduces nuclear translocation of nuclear factor kappa-B (NF-κB) protein, thereby decreasing the expression of inflammatory cytokines in cardiovascular and cerebrovascular diseases, through preventing [27]. Therefore, PARP-1 is closely related to the anti-inflammation and autophagy in treatment effects of TGP in treating CHF. Although TGP has excellent potential as a clinical therapy for CHF, its unknown mechanism limits its wider use. This study aims to explore the role and mechanism of TGP in treating CHF using animal and cell models of the disease.

## Methods and Materials

### Animal Model

TGP capsules were manufactured by Ningbo Lihua Pharmaceutical Co., Ltd., of Zhejiang Province, China, approved by the China Food and Drug Administration (approval number: H20055058), and the main active ingredient is paeoniflorin. TGP was diluted into 10- and 5-g/L suspensions (1% CMC-Na) before use, while captopril (95% purity) was obtained from Shanghai Pukang Pharmaceutical Co., Ltd., China, and diluted to 2.25 g/L (distilled water) and stored at 4 °C for future use.

By ligating the left anterior descending artery (LAD) in 10-week-old male Wistar rats (Shanghai Laboratory Animal Center, Chinese Academy of Sciences), a heart failure model was established. The rats were given 80 mg/kg ketamine and 5 mg/kg acepromazine (both from Sigma-Aldrich, St. Louis, MO, USA), and a left thoracotomy was conducted. The heart was externalized, and a Prolene 6-0 suture was used to ligate the LAD 2 mm from its origin. Sutures were passed but not knotted in the sham-operated animals. The animals were then closed in three layers following the technique. To combat bacterial infection immediately following surgery, penicillin was administered intraperitoneally at a rate of 200,000 U/day for 3 days. In the next 4 weeks, the rats were separated into six groups (*n* = 9): control group (Ctrl), sham group, model group (CHF), CHF+captopril group [CHF+Cap; CHF rats were given Cap (15 mg/kg) by gavage, once a day for 12 weeks], CHF+TGP-low dosage group [CHF+TGPlow; CHF rats were given TGP (200 mg/kg) by gavage, once a day for 12 weeks], and CHF+TGP-high dose [CHF+Caphigh; CHF rats were given TGP (400 mg/kg) by gavage, once a day for 12 weeks]. The rats in the control, sham, and CHF groups received distilled water (0.2 mL/10 g) by gavage, once a day for 12 weeks.

### Echocardiography for Cardiac Function Evaluation

After the end of the 24-h gavage, echocardiography was used for left ventricular (LV) cardiac structure and function in rats as previously reported [28]. Rats were injected with anesthesia of 3% isoflurane (1% isoflurane for maintaining). The procedure was performed by an expert operator blinded to the treatment assignment using a 20-MHz probe connected to a Vevo 770 high-frequency ultrasound system, which was used to image the LV (VisualSonics, Toronto, ON, Canada). The main indicators for analysis are ejection fraction (EF) and fractional shortening (FS).

### Blood Samples and Heart Tissues

Blood samples: After a 48-h echocardiography, animals were deprived of food and water for 3 h before weighting and blood collection. To induce anesthesia in the rats, 1% pentobarbital sodium was injected into the abdominal cavity. After immediately opening their abdominal aorta, 10–15 mL of arterial blood was taken using vacuum vasculature. The blood is allowed to stand until coagulation occurs to collect serum. Next, centrifugation was performed at 3000 rpm for 15 min at 4 °C, and the serum was transferred to 500-μL EP tubes and stored at −80 °C for future research.

Heart tissues: The auricular appendix was removed from the thoracic cavity of rats. The abdominal aorta was perfused with 0.9% saline solution (60–120 mL) until the liquid flowing out became clear in color. The rat heart was dissected and cleaned with normal saline before being dried on filter paper. The weight of the heart was determined using an electronic balance. Fresh heart tissue (taken from 3 rats per group) was fixed using 2.5% glutaraldehyde, while other hearts (taken from 3 rats per group) were rapidly frozen in liquid nitrogen and stored in freezing tubes at −80 °C. An additional set of hearts was preserved using 4% paraformaldehyde fixative for detecting gene and protein expression.

### Observation of Cardiac Histopathology and Cell Apoptosis

Observation of pathological changes in myocardial tissue was done using hematoxylin-eosin (H&E) staining, myocardial fibrosis was observed using Masson’s trichrome staining, and cell apoptosis was measured using terminal dUTP nick end labeling (TUNEL) assay. The hearts of the rats in each group were extracted, fixed in 4% paraformaldehyde, embedded in paraffin, and sectioned into 5-μm sections. Following that, H&E staining kit (G1003, Servicebio, China), Masson’s trichrome staining kit (G1006, Servicebio, China), and TUNEL staining kit (G1507, Servicebio, China) were used for observing LV myocardial slices. According to H&E staining results, the degrees of tissue cell damage, fiber arrangement, and inflammatory cell infiltration were evaluated based on a previous report [29]. For Masson’s trichrome staining, in an average of five sections from each heart, myocardial fibrosis was reported as a proportion of fibrotic area to left ventricular area (% of LV). Under light microscopy, the number of TUNEL-positive cardiomyocyte nuclei was manually counted in total, including the LV posterior wall and septum, from each LV short-axis segment. We counted and represented as a percentage of total cardiomyocytes in a specific LV region only the nuclei obviously found inside cardiomyocytes.

### Transmission Electron Microscope for Tissues

Myocardial tissues were fixed with 2.5% glutaraldehyde (111-30-8, Alfa Aesar, UK) and were cut into 1 mm × 1 mm × 1 mm pieces. After cleaning, dehydrating, embedding, slicing, and staining the tissues, they were viewed using a transmission electron microscope (TEM) (H-7650, Hitachi, Tokyo, Japan). A pathologist conducted all exams at a magnification of ×30,000. Each tissue sample was examined for its myofibrillar structure, mitochondrial membrane, and characteristic autophagic vacuoles.

### ELISA Assay

Following collection of a blood sample from rats, the concentrations of N-terminal pro-B-type natriuretic peptide (NT-proBNP) (MM-032R1), C-reactive protein (CRP) (MM-0081R1), TNF-α (MM-0180R1), IL-6 (MM-0190R1), and monocyte chemotactic protein-1 (MCP-1) (MM-0099R1) in the serum were determined using an enzyme-linked immunosorbent assay (ELISA) (MEIMIAN, China) according to the manufacturer’s instructions. The values were standardized to the quantities of cell protein in the samples.

### Cell Culture

The H9C2 rat cardiomyocytes were obtained from the Cell Bank of the Chinese Academy of Sciences (Shanghai, China) and cultured in Dulbecco’s modified Eagle’s medium (DMEM) (11885084, Gibco, USA) supplemented with 10% fetal bovine serum (FBS) (10099141C, Gibco, USA) and 100 U/mL penicillin/100 mg/mL streptomycin at 37 °C in an atmosphere of 90% air and 10% CO_2_; the medium was replenished every 2 days; and the cells were digested with 0.05% trypsin when the density of the cells reached 80–90%. H9C2 cells (1 × 10^5^ cells/mL) were seeded in a 25-cm^2^ culture flask (430639) or 6-well (3506) or 96-well culture plates (353936) and treated identically to those used in the subsequent investigations. They were purchased from Corning Incorporated, NY, USA.

### Transfection

H9C2 cells (1 × 10^5^ cells/mL) were transfected with poly(ADP-ribose)polymerase 1 (PARP-1) siRNA (sc-29437, Santa Cruz Biotechnology, CA, USA) or a negative control in Opti-MEM Medium (31985062, Invitrogen, USA) in order to decrease PARP-1 expression. H9C2 cells were transfected with the plasmids pcDNA3.1-PARP-1 or pcDNA3.1-vector (GeneChem Co., Ltd., Shanghai, China) in serum-free media. Lipofectamine 2000 (11668019, Invitrogen, USA) was used to transfection. The experiments were carried out 48 h following the transfection.

### Cell Treatment

Adriamycin (ADR) treatment induces myocardial cell damage leading to heart failure [30]. Therefore, we used ADR-incubated cells to induce myocardial cell injury and observed the protective effect of TGP on myocardial cells. Four major experimental groups were established in the current investigation: (i) control group: DMEM, (ii) ADR group: adriamycin treatment (2 μM) for 48 h, (iii) ADR+TGP group: TGP treatment (10 μg/mL) for 12 h followed by ADR treatment (2 μM) for 48 h, (iv) ADR+TGP+OE-PARP-1 group: TGP treatment (10 μg/mL) for 12 h followed by ADR treatment (2 μM) of pcDNA3.1-PARP-1 transfected H9C2 cell for 48 h.

### Western Blot

Total protein from the treated cells and tissues was extracted using RIPA buffer containing 1 mmol/L PMSF (P0013D, Beyotime, China) and quantified using the BCA Protein Assay Kit (pc0020, Solarbio, China). SDS-PAGE was used to separate the protein bands, which were then transferred to a polyvinylidene difluoride (PVDF) membrane for analysis (10600023, GE Healthcare Life, USA). After being blocked with 5% bovine serum albumin (4240GR100, BioFroxx, Germany) in phosphate-buffered saline with Tween (PBST) for 1 h, the PVDF membrane was incubated with primary antibodies including cleaved caspase-3 (AF7022, Affinity, USA), TNF-α (AF7014, Affinity, USA), IL-6 (DF6087, Affinity, USA), NF-κB (AF5006, Affinity, USA), PARP-1 (ab32064, Abcam, UK), and GAPDH (AF7021, Affinity, USA) overnight at 4 °C and then with the secondary antibody for 1 h at room temperature before being exposed to the secondary antibody. The signals were determined by the Amersham Prime ECL Plus Detection system (Pittsburgh, PA).

### Flow Cytometry Analysis

Flow cytometry was used to identify apoptosis. To summarize, after collecting the cells processed according to grouping requirements using EDTA-free trypsin (BL526A, Biosharp, China), they were washed twice with PBS and filtered through a 300-mesh nylon to get a single-cell suspension. The cell density is 2 × 10^5^ cells/mL. Then, the Annexin V-FITC apoptosis detection kit (APOAF-20TST, Sigma, Japan) was used to determine the apoptosis rates. Annexin V-FITC (5 μL) was added into a 195-μL cell suspension to incubate for 10 min at 25 °C. Cells were washed and resuspended in 190 μL binding buffer (1×). After adding 10 μL propidium iodide, samples were performed to flow cytometry (Accuri C6, BD, NJ, USA).

### CCK-8 Assay

Cell viability was determined according to the manufacturer’s procedure using Cell Counting Kit-8 (CCK-8, Beyotime, Shanghai, China). Cells were seeded and cultured at a density of 5 × 10^3^/well in 100 μL of medium in 96-well microplates (Corning, USA). Cells were incubated sequentially in complete medium containing ADR, TGP, or no treatment according to their designated groupings. After treatment for 72 h, 10 μL of CCK-8 was added to each well and then cultured for 2 h. All experiments were performed in triplicate. The absorbance at 450 nm was determined using a microplate reader (Bio-Rad, Hercules, CA, USA) with blank wells. The absorbance of cells was used to quantify their proliferation.

### Real-Time Polymerase Chain Reaction (RT-PCR)

The TRIzol extraction method was used to extract RNA from cells and tissues (Invitrogen, CA, USA). Following that, cDNA was evaluated by RT-PCR using iQ™ SYBR Green Supermix (Bio-Rad Laboratories, CA, USA). The sequences of specific primers are as follows: PARP-1-F: ACCACGCACAATGCCTATGA, PARP-1-R: AGCAGTCTCCGGTTGTGAAG; GAPDH-F: TTCCTACCCCCAATGTATCCG, GAPDH-R: CATGAGGTCCACCACCCTGTT. The real-time PCR system was used for amplification, detection, and data processing (Bio-Rad Laboratories). The real-time program featured a 1.5-min denaturation phase at 95 °C, 40 cycles at 95 °C for 15 s, and 30 s at 60 °C. Each sample was analyzed in triplicate, and each experiment was performed three times. The 2^−ΔΔCt^ method was used to analyze the relative changes in gene expression.

### Immunofluorescence

H9C2 cells were pretreated for 30 min with PBS or remifentanil and then treated differently. Following that, the cells were fixed in 4% paraformaldehyde and permeabilized in PBS containing 0.1% Triton X-100. After 30 min of blocking with BSA, the samples were incubated overnight at 4 °C with primary antibody rabbit anti-NF-κB p65 (1: 100, #8242, Cell Signaling Technology, USA). As a secondary antibody, Alexa 488-conjugated goat anti-rabbit IgG (1: 500, #4414S, Cell Signaling Technology, USA) was utilized. To seal the coverslip, a drop of ProLong Gold Antifade Reagent containing 4′,6-diamidino-2-phenylindole (DAPI; Vector Laboratories, CA, USA) was utilized. Laser scanning confocal microscopy was used to obtain the images (LSM 710, Zeiss, Germany). Image-Pro Plus 6.0 was used to examine the data.

### Transmission Electron Microscopy for Cellular Ultrastructure Analysis

As previously reported [31], we used electron microscopy to observe the cellular ultrastructure. Briefly, the cell culture medium was replaced with 2.5% glutaraldehyde anterior fixative to fix for 2 h at 25 °C and then 1% osmic acid was used to further fix for 2 h at 4 °C. After sequential dehydration, washing, and embedding, the cell samples were cut into ultrathin slices (100 nm) using an ultramicrotome and counterstained with 0.3% uranium acetate and lead nitrate, and the number of autophagosomes was evaluated using a TEM (H-7650, Hitachi, Tokyo, Japan).

### Statistical Analysis

All data were analyzed using SPSS 25 (IBM, USA) and presented as mean ± standard deviation (SD). GraphPad Prism 9 (GraphPad Software, USA) was used to display the results. Multi-group comparisons were performed by one-way analysis of variance (ANOVA) followed by Tukey post-hoc test for homogenous variance or Dunnett’s T3 for non-homogenous variance. Additionally, Kruskal-Wallis test was used for analyzing data that did not follow a normal distribution. *P* < 0.05 was considered as statistically significant.

## Results

### TGP Improved the Heart Function and Serum Inflammatory Levels in CHF Rats

**Fig. 1:**
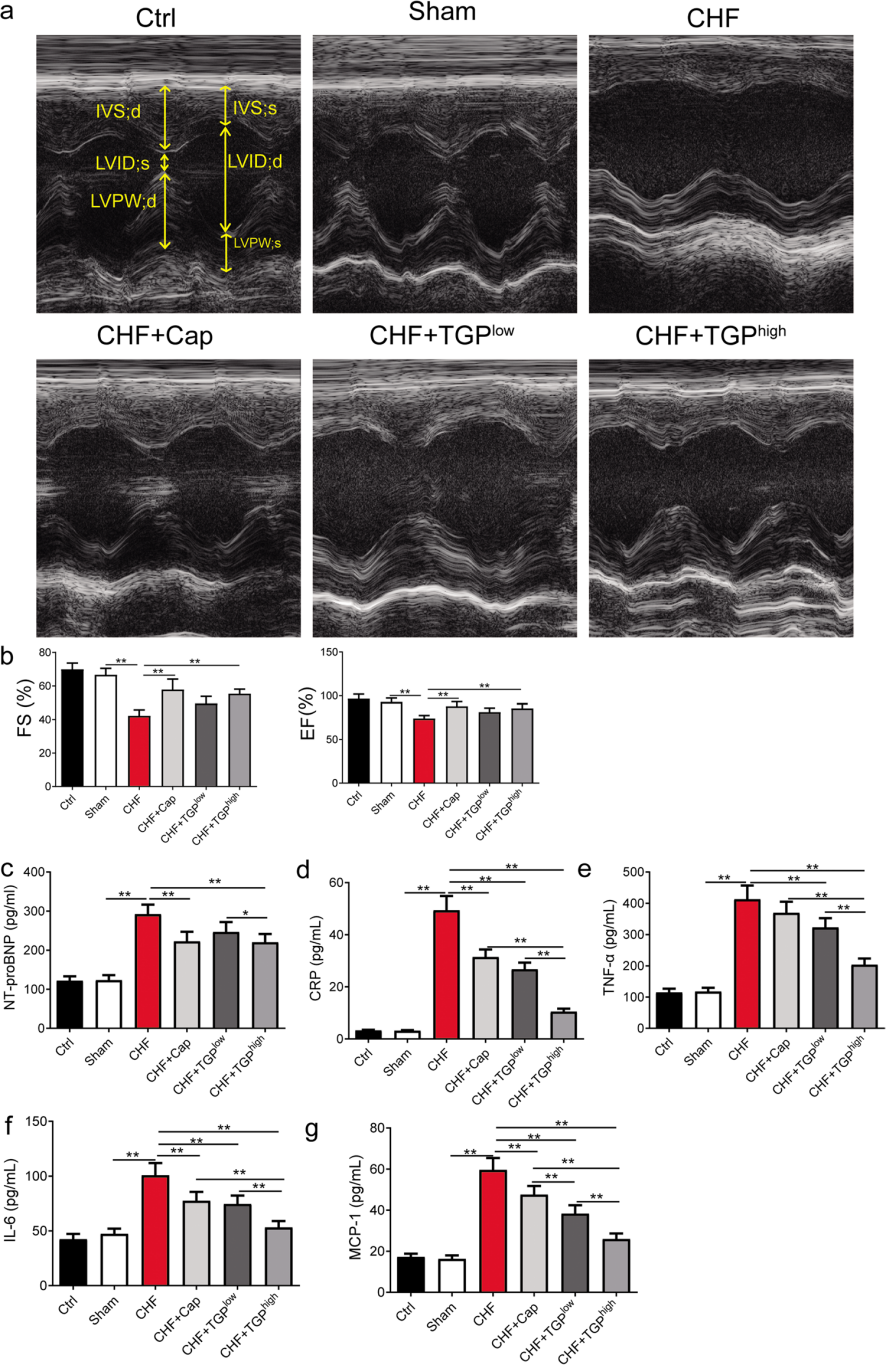
TGP improved the heart function in CHF rats. **a** Representative echocardiography images. **b** The ejection fraction (EF) and fractional shortening (FS) are echocardiographic parameters in rats (*n* = 5). Additionally, ELISA analysis of the levels of **c** NT-proBNP, **d** CRP, **e** TNF-α, **f** IL-6, and **g** MCP-1 in rats with ligating the left anterior descending artery, 15 mg/kg Cap, or 200/400 mg/kg TGP and the control. The data are presented as mean ± SD. Horizontal line segments with “*” indicates *P* < 0.05 and “**” indicates *P* < 0.01, and each end of the segment corresponds to the respective group. Cap: captopril; Ctrl: control; CHF: chronic heart failure; CRP: C-reactive protein; NT-proBNP: N-terminal pro-B-type natriuretic peptide; MCP-1: monocyte chemotactic protein-1; TGP: total glucosides of paeony; IL-6: interleukin-6; TNF-α: tumor necrosis factor-α

We used high-resolution echocardiography to assess heart function in all groups. M-mode echocardiograms were performed from a short axis, which are shown in Fig. 1a; the FS and EF were used to evaluate LV function (Fig. 1b). There was no significant difference in rats’ LV function between the ctrl group and sham group (Fig. 1b). The FS and EF values decreased in CHF rats compared to the sham group (*P* < 0.01), while Cap and TGP^low/high^ treatment raised the EF and FS percentages in CHF rats (Fig. 1b). In addition, the FS and EF values of TGP^low^- and TGP^high^-treated rats were comparable to those of the Cap therapy (*P* > 0.05) (Fig. 1b). Additionally, we performed ELISA tests to determine the levels of NT-proBNP, CRP, TNF-α, IL-6, and MCP-1 in rat serum. Rats with a ligated LAD had elevated levels of NT-proBNP, CRP, TNF-α, IL-6, and MCP-1. In CHF rats, those levels were significantly higher than those in sham rats (*P* < 0.01), while Cap and TGP^high^ therapy significantly suppressed their concentrations (*P* < 0.01) (Fig. 1c–g). TGP^low^ treatment on CHF rats significantly suppressed the CRP, TNF-α, IL-6, and MCP-1 levels (*P* < 0.01) (Fig. 1d-g). Furthermore, inhibition of TGP^high^ in the levels of CRP, TNF-α, IL-6, and MCP-1 in CHF rats was superior to Cap.

### TGP Ameliorate Myocardial Tissue Damage in CHF Rats

**Fig. 2:**
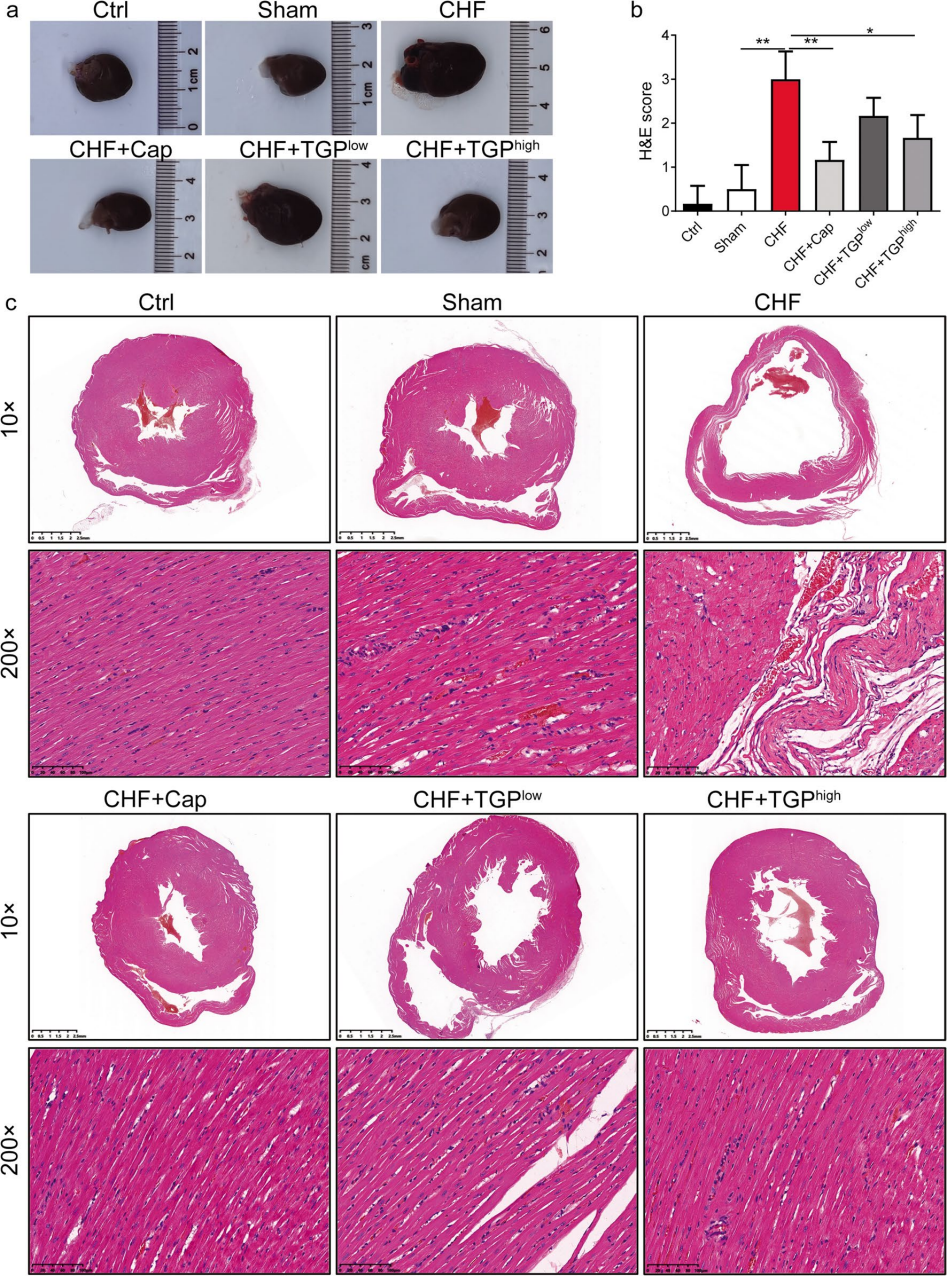
TGP ameliorate histopathological damage of hearts in CHF rats. **a** Representative photos of the heart. **b** H&E staining score and **c** representative images of the myocardial tissue from rats with ligating the left anterior descending artery, 15 mg/kg Cap, or 200/400 mg/kg TGP and them control (*n* = 6, scale bars in 10× and 200× images were 2.5 mm and 100 μm). The data are presented as mean ± SD. Horizontal line segments with “*” indicates *P* < 0.05 and “**” indicates *P* < 0.01, and each end of the segment corresponds to the respective group. Ctrl: control; CHF: chronic heart failure; Cap: captopril; H&E: hematoxylin-eosin; TGP: total glucosides of paeony

To examine TGP’s impact on the heart in vivo, CHF rats were given a dose of TGP solution (200 or 400 mg/kg day) for 12 weeks. We observed that the heart shape in the CHF rats and TGP^low^-treated rats was changed and its volume increased compared to the control and sham rats (Fig. 2a). H&E staining was used for histological examination of myocardial tissue. The results revealed that in CHF rats, the ventricular chamber was considerably enlarged, the myocardial cells showed hypertrophy with disordered arrangement, and the myocardial tissues presented much fibrosis; correspondingly, the score of H&E staining was increased compared to sham rats (*P* < 0.01) (Fig. 2b, c). The treatment of Cap or TGP^low/high^ can improve the morphology and disorder arrangement of myocardial cells, and H&E scores of the CHF+Cap and CHF+TGP^high^ groups were significantly lower than those of CHF rats (*P* < 0.05) (Fig. 2b, c). Additionally, we observed that therapy with TGP^low/high^ and Cap reduced the size of the ventricular chamber in CHF rats (Fig. 2c). Moreover, there was no significant difference in HE scores among the three treatment groups suggesting the improvement effect of Cap and TGP on tissue damage is similar.

### TGP Suppressed Cardiac Fibrosis, Apoptosis, and Autophagy in CHF Rats

**Fig. 3:**
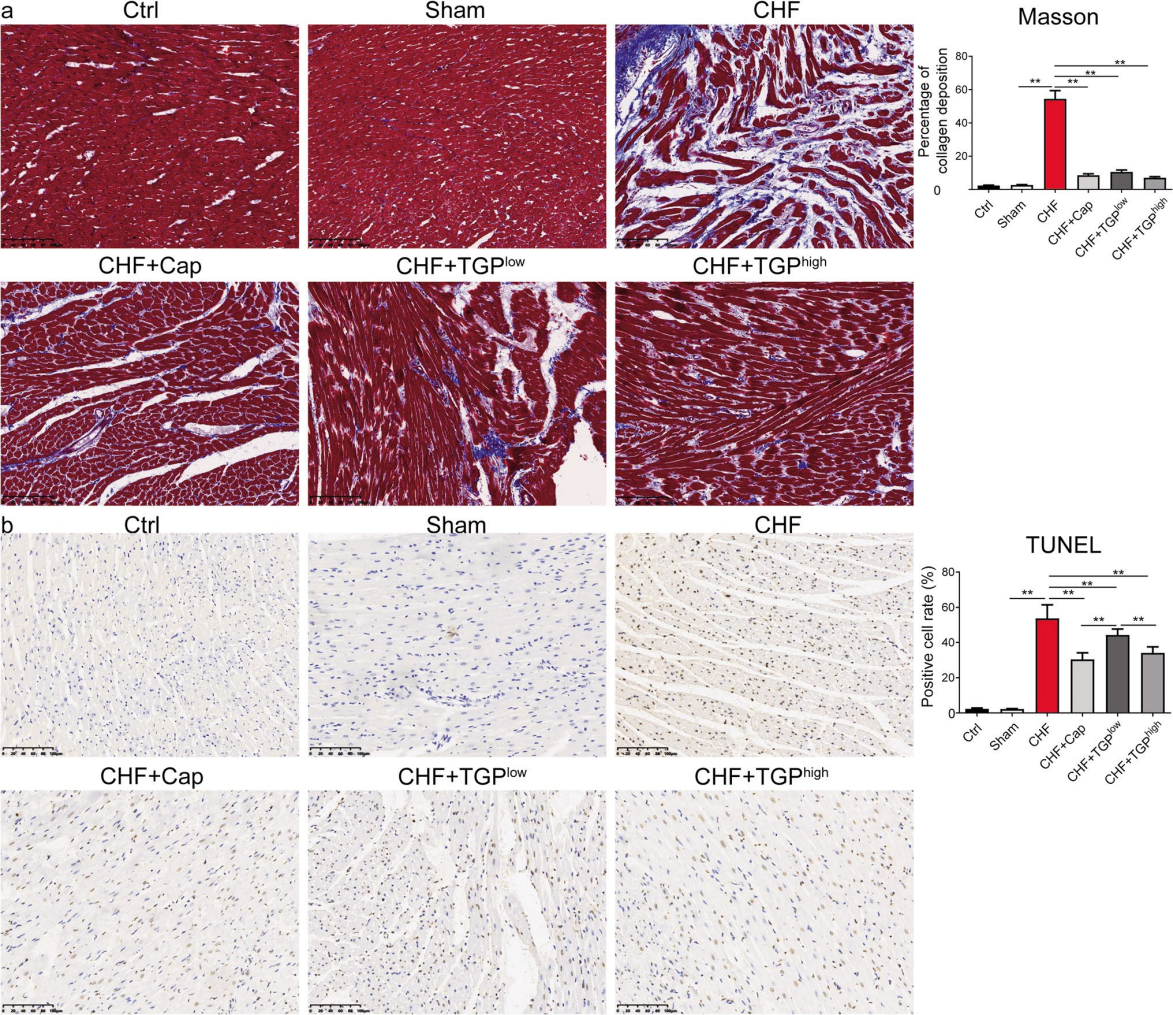
TGP suppressed cardiac fibrosis, apoptosis, and autophagy in myocardial tissues of chronic heart failure rats. **a** Masson trichrome staining of myocardial tissues was used to observe collagenous fiber (*n* = 6, 200×, scale bars = 100 μm). **b** TUNEL staining was used to observe apoptosis (*n* = 6, 200×, scale bars = 100 μm). Rate of TUNEL-positive cell to total was manually counted. The data are presented as mean ± SD. Horizontal line segments with “*” indicates *P* < 0.05 and “**” indicates *P* < 0.01, and each end of the segment corresponds to the respective group. Ctrl: control; CHF: chronic heart failure; Cap: captopril; TGP: total glucosides of paeony; TUNEL: terminal dUTP nick end labeling

**Fig. 4:**
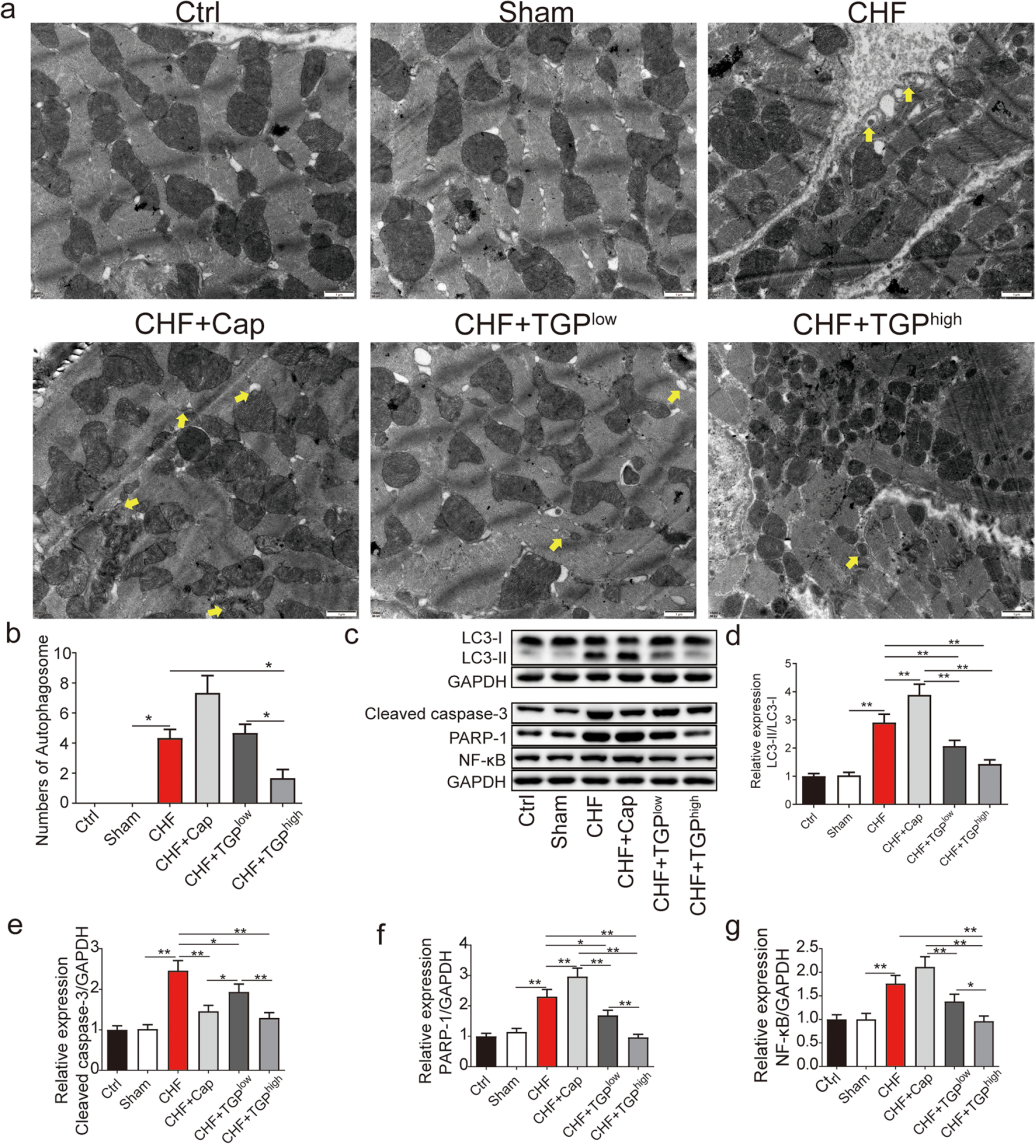
TGP affected the autophagosomes and inhibited apoptosis and autophagy related proteins. **a**, **b** Transmission electron microscopy images and numbers of myocardial autophagosomes. The arrow indicates typical autophagic autophagosomes (*n* = 3, 30,000×, scale bar = 1 μm). Western blot was used to measure protein expression (*n* = 3). **c** Representative protein bands of Western blot. The results analysis of **d** LC3-II/LC3-I, **e** cleaved caspase-3, **f** PARP-1, and **g** NF-κB protein expression were shown that TGP treatment on CHF rats inhibited those protein expression. The data are presented as mean ± SD. Horizontal line segments with “*” indicates *P* < 0.05 and “**” indicates *P* < 0.01, and each end of the segment corresponds to the respective group. Ctrl: control; CHF: chronic heart failure; Cap: captopril; NF-κB: nuclear factor kappa-B; PARP-1: poly(ADP-ribose)polymerase 1; TGP: total glucosides of paeony

Cardiac remodeling is a key foundation for the development of heart failure, and cardiac fibrosis is an important manifestation of cardiac remodeling [32]. In Masson’s trichrome staining, the collagenous fiber is blue, and the results revealed an abnormal amount of collagen deposition in the myocardium of CHF rats (Fig. 3a). Cap and TGP treatment had a substantial effect in reducing deposition suggesting that Cap and TGP can prevent myocardial fibrosis (*P* < 0.01) (Fig. 3a). Research shows that regulating autophagy and apoptosis can prevent heart remodeling [33]. Therefore, we used TUNEL staining and TEM to evaluate the autophagy and apoptosis (Figs. 3b and 4a, b). The number of TUNEL-positive cells was significantly increased in the myocardium of CHF rats (*P* < 0.01), while Cap and TGP treatment reduced the quantity of apoptotic cells (*P* < 0.01) (Fig. 3b). Additionally, using TEM, the myofibrillar structure and mitochondrial membrane integrity were preserved in the control and sham groups with no autophagosomes, but the autophagosome number is significantly higher than that of sham rats (*P* < 0.05) (Fig. 4a). The autophagosome number in rats with TGP^high^ treatment was significantly reduced (*P* < 0.01). In Cap- and TGP^low^-treated rats, the autophagosome number showed no difference compared to the CHF rats (*P* > 0.05) (Fig. 4a). The LC3-II/LC3-I ratio was increased in CHF rats compared to sham rats (*P* < 0.01); Cap treatment increased the LC3-II/LC3-I ratio in CHF rats (*P* < 0.01), while TGPlow/high treatment inhibited it (*P* < 0.01) (Fig. 4c, d).

Additionally, treatment with Cap and TGP decreased the increase in cleaved caspase-3 in the CHF group (Fig. 4c, d). Moreover, PARP-1 and NF-κB expressions were elevated in the CHF group compared to the sham group (*P* < 0.01) (Fig. 4c, e, f). Gavage with Cap increased the PARP-1 expression in CHF rats, while TGP^low/high^ both decreased it (*P* < 0.05) (Fig. 4e). Furthermore, treatment with TGP^high^ significantly reduced the NF-κB protein level in myocardial tissue of CHF rats (*P* < 0.01) (Fig. 4c, f). We found the regulation of TGP on PARP-1 and NF-κB expression.

### PARP-1 Overexpression Promoted the Nuclear Translocation of NF-κB to Increase the Expression of LC3-II/I, IL-6, and TNF-α

**Fig. 5:**
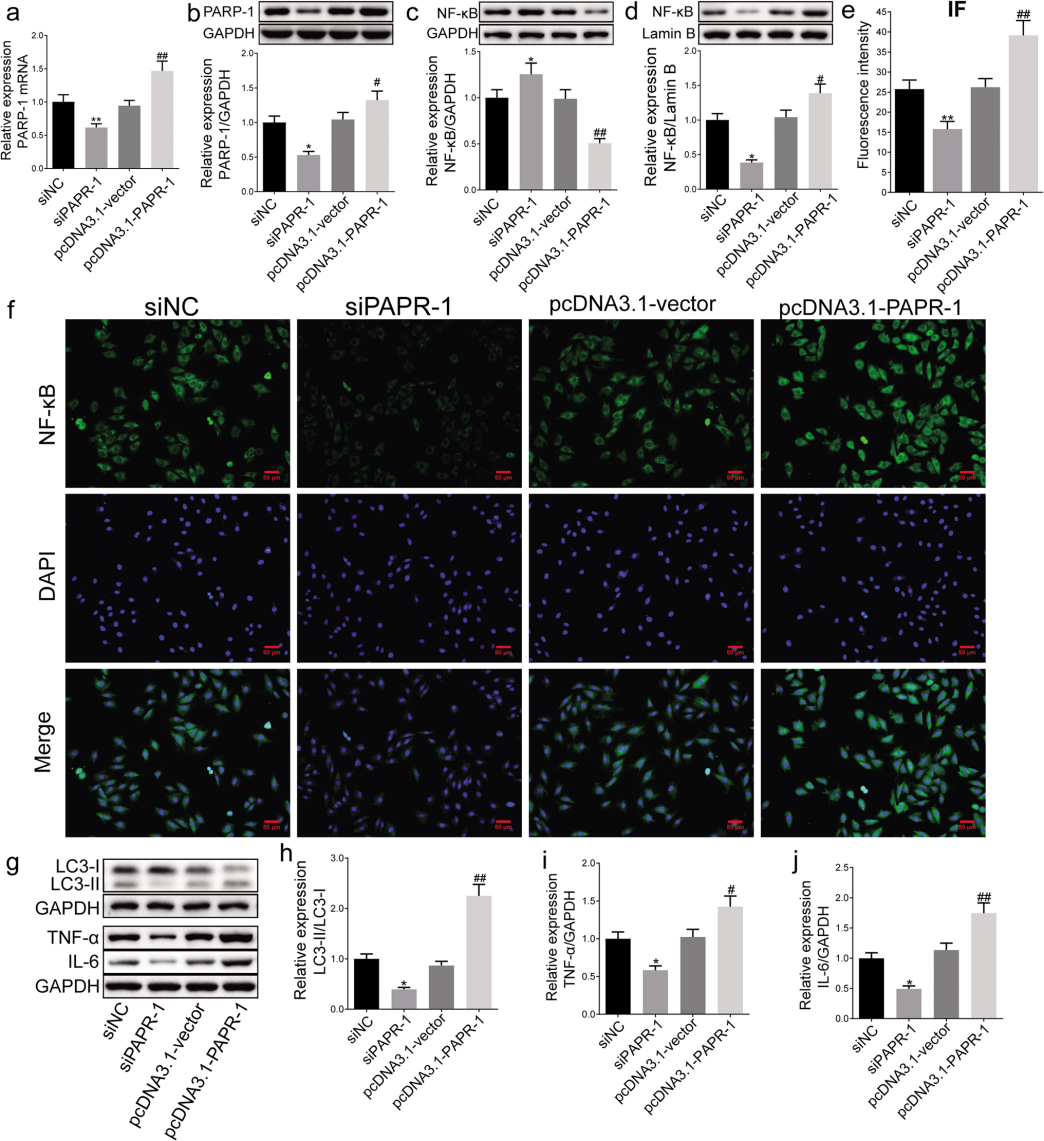
PARP-1 promoted the nuclear translocation of NF-κB to decrease the expression of LC3, IL-6, and TNF-α in cells with RARP-1 knockdown and overexpression. Real-time polymerase chain reaction (a) and Western blot (b) analysis were used to measure PARP-1 expression in H9C2 cells with PARP-1 knockdown and overexpression (*n* = 3). **c**, **d** Western blot analysis was used to detect the NF-κB level in cytoplasm and nucleus. **e** Fluorescence intensity of immunofluorescence staining was detected to observe the NF-κB expression in cells (*n* = 3, 200×, scale bars = 100 μm). **f** Representative photos of NF-κB immunofluorescence. Western blot used to measure LC3-II/I, TNF-α, and IL-6. **g** Representative protein bands and results of the expression of **h** LC3-II/LC3-I, **i** TNF-α, and **j** IL-6. The data are expressed as mean ± SD. ^*^P < 0.05 and ^**^P < 0.01 compared with the siNC group, ^#^*P* < 0.05 and ^##^*P* < 0.01 compared with vector group. IL-6: interleukin-6; LC3: light chain 3; NF-κB: nuclear factor kappa-B; PARP-1: poly(ADP-ribose)polymerase 1; TGP: total glucosides of paeony; TNF-α: tumor necrosis factor-α

To explore the regulatory effect of PARP-1 on NF-κB in cardiomyocytes, PARP-1 knockdown and overexpression were successfully performed in H9C2 cells (Fig. 5a, b). PARP-1 overexpression enhanced nuclear NF-κB expression and decreased cytoplasmic NF-κB expression, but PARP-1 knockdown had the opposite effect (Fig. 5c, d). Additionally, the NF-κB protein signal intensity was significantly inhibited in PARP-1 knockdown, while PARP-1 overexpression enhanced it compared to their control (Fig. 5e, f). Furthermore, we discovered that silencing PARP-1 decreased the expression of LC3, IL-6, and TNF-α, but overexpression of PARP-1 enhanced their production (Fig. 5g–j).

### TGP Inhibited Autophagy and Apoptosis of Cardiomyocytes by Inhibiting PARP-1 and the NF-κB Pathway

**Fig. 6:**
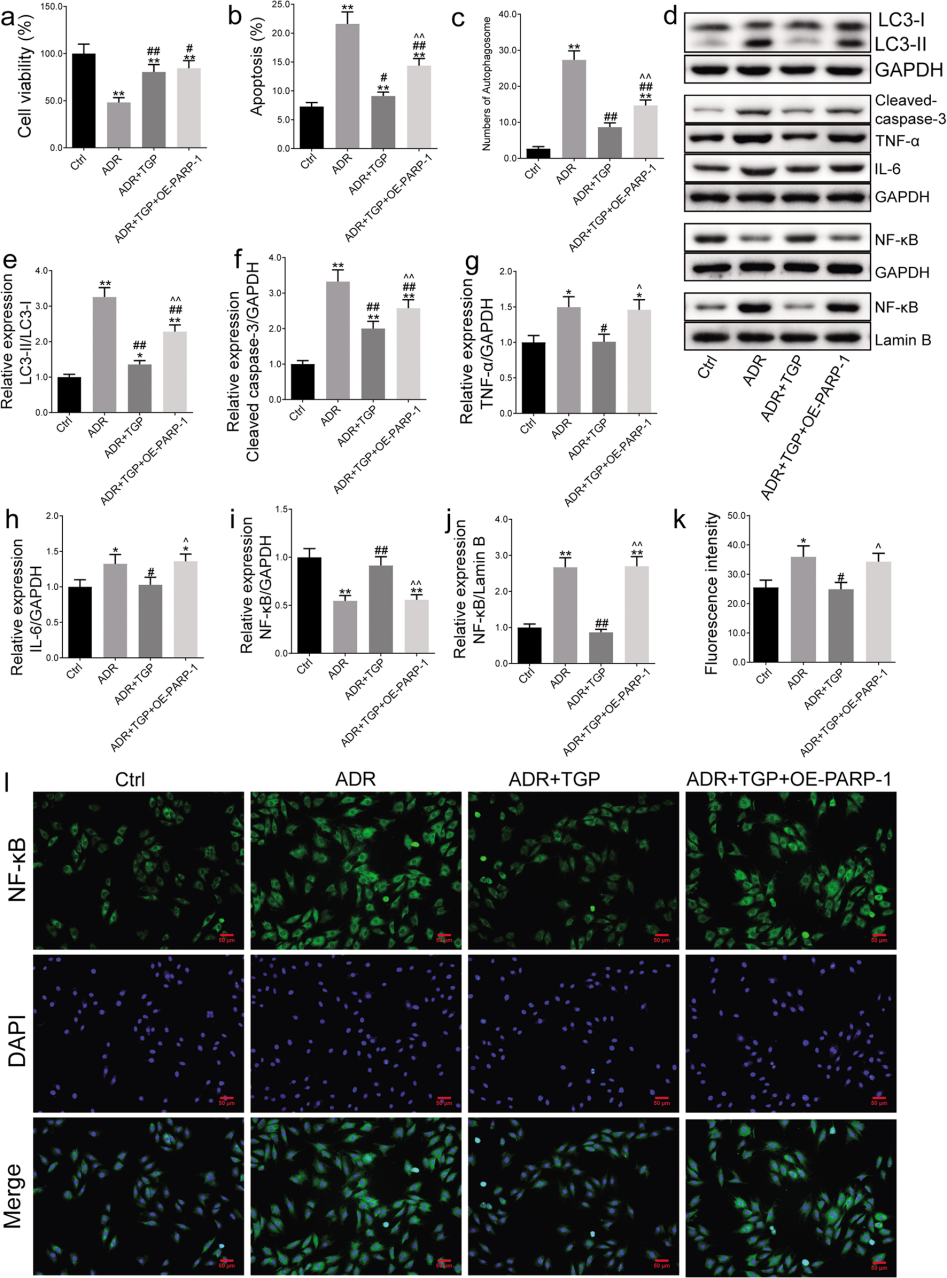
TGP inhibited autophagy, apoptosis, and regulated related-protein expression in H9C2 cells with PARP-1 overexpression. Adriamycin treatment on H9C2 cells with PARP-1 overexpression were used to establish myocardial injury model observing the mechanism of TGP on PARP-1. **a** Cell viability was measured using CCK-8 analysis. **b** Cell apoptosis rate was measured using flow cytometry analysis. (c) Transmission Electron Microscope for cellular ultrastructure analysis. **d** Representative protein bands of Western blot. The expression of **e** LC3-II/LC3-I, **f** cleaved caspase-3, **g** TNF-α, and **h** IL-6 were measured using Western blot. **i**, **j** Western blot analysis of NF-κB in cytoplasm and nucleus. **k** Immunofluorescence staining was used to observe the expression of NF-κB. Fluorescence intensity of immunofluorescence was decreased in TGP treatment H9C2 cells. **i** Representative photos of NF-κB immunofluorescence. The data are expressed as the mean ± SD. ^**^P < 0.01 compared with ctrl group, ^#^*P* < 0.05 and ^##^*P* < 0.01 compared with the ADR group, ^^^^*P* < 0.01 compared with the ADR+TGP group. ADR: adriamycin; Ctrl: control; IL-6: interleukin-6; LC3: light chain 3; NF-κB: nuclear factor kappa-B; PARP-1: poly(ADP-ribose)polymerase 1; TGP: total glucosides of paeony; TNF-α: tumor necrosis factor-α

ADR treatment can induce myocardial cell damage leading to heart failure [30]. Therefore, we used ADR-incubated cells to establish a myocardial cell injury model and observed the protective effect of TGP on cardiomyocytes. ADR treatment reduced cell viability and apoptosis in H9C2 cells (*P* < 0.01) (Fig. 6a, b; SI Fig. 1a) suggesting that TGP incubation can alleviate the cell injury that is induced by ADR. However, PARP-1 overexpression partially antagonized the TGP effect on apoptosis in ADR-treated H9C2 cells (Fig. 6b; SI Fig. 1a). Additionally, TEM analysis revealed that the ADR group had a greater number of autophagosomes than the control, while TGP incubation decreased the number of autophagosomes (*P* < 0.01) (Fig. 6c; SI Fig. 1b). Furthermore, overexpression of PARP-1 partially antagonized the inhibition effect of TGP on ADR-treated cells (Fig. 6c; SI Fig. 1b). In addition, ADR increased the ratio of LC3-II/LC3-I, cleaved caspase-3, IL-6, and TNF-α (*P* < 0.05), whereas TGP blocked these effects (*P* < 0.05) (Fig. 6d–h). PARP-1 overexpression partially antagonized TGP’s inhibitory impact on these proteins (*P* < 0.05) (Fig. 6d–h). Besides, we found that TGP inhibited nuclear translocation of NF-κB in cells with ADR incubation, which were observed using western blot assay (*P* < 0.01) (Fig. 6i, j). Results of cellular immunofluorescence have shown that ADR treatment enhanced signal strength of NF-κB, while TGP reduced it (*P* < 0.01) (Fig. 6l). Overexpression of PARP-1 partially reversed the effects of TGP on NF-κB in cells with ADR treatment (Fig. 6i–l).

## Discussion

Our main findings indicate that treatment with TGP improved heart function in CHF rats. Additionally, TGP inhibited both autophagy and apoptosis of cardiomyocytes as well as PARP-1 and the NF-κB pathway to prevent the progression of CHF. Several factors contribute to cardiac remodeling and exacerbate CHF, such as myocardial hypertrophy and fibrosis, aberrant myocardial cell apoptosis and autophagy, and inflammatory response [34, 35]. We simulated CHF in rats by performing LAD ligation, which is a common procedure for simulating CHF [36]. Additionally, to determine the ventricular failure status of rats, we measured their NT-pro-BNP levels (a clinical biomarker for heart failure diagnosis [37]), inflammatory factors, cardiac impulse, atrioventricular diameter, and hemodynamics. Inflammatory factors tend to be elevated in CHF patients compared to healthy individuals; increased levels of inflammatory markers (CRP, TNF-α, IL-6, MCP-1) and NT-pro BNP have been found to correlate with the CHF severity [38, 39]. Consistent with clinical results, our results showed that LAD ligation induced heart failure, characterized by reduced FS, EF, and cardiac chamber expansion.

To evaluate TGP’s therapeutic efficacy in treating CHF rats, we used captopril-treated CHF rats as a positive control group. Our study revealed that a high dose of TGP effectively improved myocardial damage, delayed ventricular remodeling, controlled inflammatory responses, and reduced myocardial fibrosis. The effects of TGP in CHF rats were comparable to those of captopril-treated CHF rats. However, unlike captopril, TGP prevented autophagy. Captopril has been shown to suppress autophagy at doses more than 50 mg/kg [40, 41], but in this study, we used a low-dose captopril (15 mg/kg) that may induce protective autophagy. Autophagy protects cells from external stimuli and helps them escape apoptosis triggered by pathogenic damage or stress [42]. Autophagy is tightly linked to cardiomyocyte self-metabolism and is involved in ventricular remodeling [43]. Excessive autophagy can also induce myocardial apoptosis and fibrosis, leading to left ventricular dysfunction [44]. Our study indicates that TGP may inhibit excessive autophagy in the myocardium of CHF rats, which may be one of the molecular processes behind TGP’s effectiveness in treating heart failure.

We observed a significant overexpression of PARP-1 and NF-κB in the myocardial tissue of CHF rats. PARP-1 plays a crucial role in the progression of various cardiovascular diseases [45, 46]. Its activation exacerbates myocardial ischemia-reperfusion damage by promoting autophagy [23]. Moreover, the increased expression of PARP-1 may boost the expression and nuclear translocation of NF-κB p65, leading to elevated levels of TNF-α and IL-6, thereby initiating an inflammatory response [47]. In addition, activation of NF-κB enhances cell autophagy and the inflammatory response increasing cell apoptosis [48]. Our results indicate that PARP-1 represents a viable therapeutic target in the treatment of CHF. Interestingly, we found that low-dose captopril can increase PARP-1 and NF-κB expression and might increase protective autophagy through increased PARP-1 expression. We speculate that increased PARP-1 expression may enhance the release of inflammatory substances by increasing NF-κB expression, which may be the primary cause for low-dose captopril treatment on CHF without inhibiting TNF-α. Our study revealed that PARP-1 overexpression promoted the nuclear translocation of NF-κB and increased the expression of LC3, TNF-α, and IL-6. Conversely, TGP decreased the expression of PARP-1 and NF-κB, indicating that the anti-heart failure action of TGP is mediated by modulation of the PARP-1 and the NF-κB signaling pathway. Overexpression of PARP-1 antagonized TGP’s protective effect. To summarize, TGP suppressed cell apoptosis, autophagy, and inflammatory response induced by CHF via inhibiting the PARP-1 and the NF-κB signaling pathway.

However, there are limitations to this study, particularly regarding the use of TGP as a mixture containing paeoniflorin. Further investigation is necessary to compare the effectiveness of the paeoniflorin, etc., components and TGP for myocardial protection. Additionally, determining the effective dose threshold for clinical application requires further research into dose groups and TGP toxicology. In order to enhance understanding of TGP’s therapeutic capacity for cardiac failure, it would also be beneficial to utilize a larger number and greater variety of cardiac failure models. In conclusion, the data imply that autophagy activation may contribute to ventricular remodeling in patients with CHF. Treatment with TGP might serve a heart-protective role in CHF, presumably via suppressing the autophagy process. Further study is needed to elucidate the involvement of autophagy and the PARP-1/NF-κB signaling pathway in the TGP treatment for CHF. This investigation may result in the development of innovative treatment options for people with congestive heart failure.

### Supplementary Information

ESM 1 **SI Fig.** **1** Characteristic images of flow cytometry for cell apoptosis and Transmission Electron Microscope for cellular ultrastructure analysis (DOCX 1922 kb)

## Data Availability

The datasets generated/analyzed during the current study are available.
