# Supplementary material for: Total Glucosides of Paeony Ameliorate Myocardial Injury in Chronic Heart Failure Rats by Suppressing PARP-1
Source: J Cardiovasc Transl Res. 2023 Oct 13;17(2):388–402. doi: 10.1007/s12265-023-10440-3 (PMC11052853; doi:10.1007/s12265-023-10440-3)
Supplement: Supplementary file 1 — SI Fig. 1 Characteristic images of flow cytometry for cell apoptosis and Transmission Electron Microscope for cellular ultrastructure analysis (DOCX 1922 kb) [file 12265_2023_10440_MOESM1_ESM.docx]

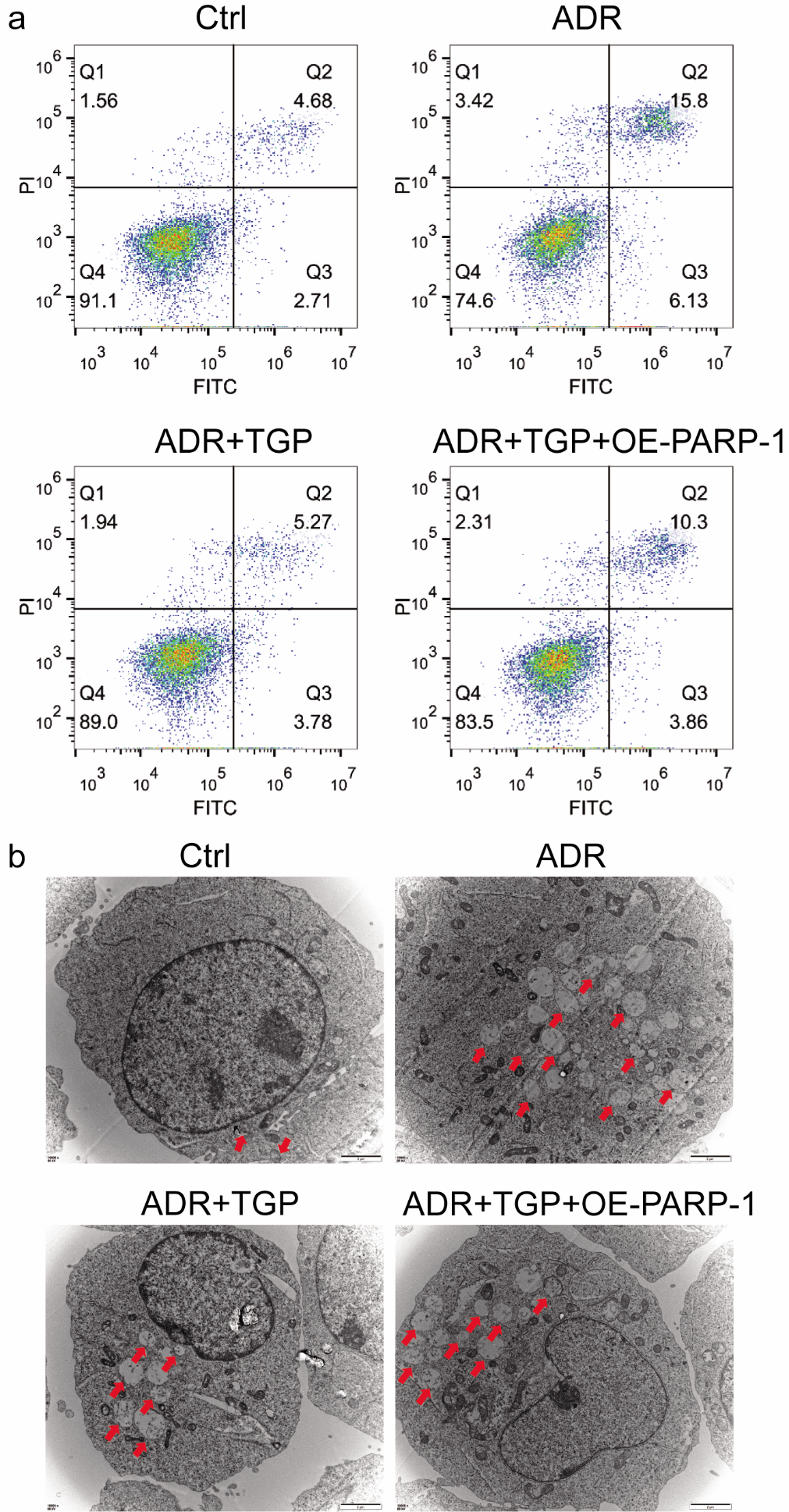


**Fig. 1** Characteristic images of (a) flow cytometry for cell apoptosis and (b) transmission electron microscope for cellular ultrastructure analysis.
